# The Territorial Medical Officers

**Published:** 1918-03-30

**Authors:** 


### THE TERRITORIAL MEDICAL OFFICERS.

It is admitted, we believe by everybody, that the stern demand of the war has fallen with special hardship on the medical officers of the Territorial regiments. At the very commencement of hostilities these officers, of course, were mobilised, and in many instances with very limited opportunities to make provision for the conduct of their practices in their absence. Colleagues who remained in civil life have, at least in some measure, done what was possible in this direction, but as months have passed into years patients have become detached from old associations, and it is literally true to say that many of these officers will return to find that their usual means of livelihood have entirely disappeared. When they are demobilised their Army pay, of course, will cease, and they will be compelled to begin their work all over again. The majority of them, it must be remembered, are not young men in the military sense of the term. They have been in practice for a number of years, have accepted obligations in the shape of rent and other charges, and have given hostages to fortune in the shape of wives and families. Moved by various motives, amongst which patriotism may surely find a place, they accepted commissions in Territorial regiments, and they found on that terrible day in August 1914 that their bond carried them away from home and work to the duties and obligations of the soldier. Perhaps to many of them, as to the rest of us, war had seemed a very unlikely and remote possibility. But the possibility realised itself, and it has meant for many of our Territorial medical officers enormous sacrifices, the full measure of which has yet to be displayed. The officers themselves can say little openly, as they are bound by official regulations. All the more incumbent is it on their civil colleagues to realise the true position and to bring such realisation home to the public conscience and memory.

We readily allow that something has been done—under the influence of a Committee which reported in September last—to redress some of the grievances of the Territorial medical officers. The most severe of these hardships, as stated above, cannot be redressed. It is part of the hard fortune of war. But on the question of payment something ought to have been done, and this step was specifically advised by the Committee just mentioned. The Army Council, however, has negatived this recommendation, and in existing circumstances an appeal to Parliament is by no means a promising prospect. Hence it remains true that the officer who was sufficiently patriotic to train himself to defend his country prior to the war, and who is relatively a senior in his profession, is less well treated than the newly qualified man who by compulsion or choice has taken service in the new armies since the war began. There are, too, niceties of rank and promotion which also are not in the favour of the Territorial officer, and which must necessarily add to his sense of unfair treatment. We wish we could see some prospect of improvement in all these directions, and we agree that the position ought to be kept in the public view. After the decision of the Army Council, however, there is little chance of any early reconsideration of the question, though we believe that later something may be done in this direction. The profession itself has some obligations in the matter, and these ought to be met in a generous and large-hearted spirit. As sacrifice there has been and must be, it ought, so far as possible, to be equally distributed.

